# Cancer screening uptake by women from India’s largest state Uttar Pradesh: district-wise analysis from the fifth round of National Family Health Survey (2019–2021)

**DOI:** 10.3332/ecancer.2024.1742

**Published:** 2024-08-20

**Authors:** Priyal Chakravarti, Kamalesh Kumar Patel, Atul Budukh, Divya Khanna, Pankaj Chaturvedi, Satyajit Pradhan, Rajesh Dikshit, Rajendra Badwe

**Affiliations:** 1 Centre for Cancer Epidemiology, Tata Memorial Centre, Navi Mumbai, 410210, India; 2 Clinical Research Unit, All India Institute of Medical Sciences, New Delhi, 110029, India; 3 Homi Bhabha National Institute, Training School Complex, Mumbai, 400094, India; 4 Department of Preventive Oncology, Mahamana Pandit Madan Mohan Malaviya Cancer Centre, Homi Bhabha Cancer Hospital, Tata Memorial Centre, Varanasi, 221005, India; 5 Department of Surgical Oncology, Tata Memorial Hospital, Mumbai, 400012, India; 6 Department of Radiation Oncology, Mahamana Pandit Madan Mohan Malaviya Cancer Centre, Tata Memorial Centre, Varanasi, 221005, India; a https://orcid.org/0000-0003-2163-796X; b https://orcid.org/0000-0003-1209-9936; c https://orcid.org/0000-0001-6723-802X; d https://orcid.org/0000-0001-7856-8059; e https://orcid.org/0000-0002-3520-1342; f https://orcid.org/0000-0003-0376-6418; g https://orcid.org/0000-0003-4830-0486; h https://orcid.org/0000-0002-0480-2831

**Keywords:** early detection of cancer, cervical cancer, breast cancer, oral cancer, rural, India

## Abstract

The Government of India (GOI) has launched a nationwide cervical, breast and oral cancer prevention and control program. However, the fifth round of the National Family Health Survey (NFHS-5), a nationwide survey conducted by the Ministry of Health and Family Welfare (MoHFW), GOI, has shown concerning results on screening uptake by both men and women across India. This study was conducted to describe the uptake of cancer screening by women residing in Uttar Pradesh (UP), the largest state of India. We analyzed NFHS-5 data available in public domain to determine the number of women (aged 30-49 years) participating in cancer screening across the 71 districts in UP state. We utilized population projections for the year 2021 provided by the population projections for India and states for calculating the number of women. The district-wise estimation was done using a projection of district-level annual population.

Although the GOI has made screening available for common cancers, NFHS-5 results indicated that the screening uptake among women aged 30-49 years is a cause for concern. The data revealed less than 1% of women underwent screening, and some of the districts showed no screening uptake. GOI has laid down a framework for cancer screening; however, poor participation among women calls for research to understand the barriers to cancer screening and to develop interventions to address these barriers.

## Introduction

With an estimated 10 million deaths in the year 2020 – or almost one in six – cancer remains a leading cause of death globally [1]. According to the Global Cancer Observatory, India accounted for 7.5% and 9.4%, respectively, of global cancer incidence and mortality [2]. Furthermore, it is projected that the cancer burden in India will increase by 12.8% in the year 2025 compared to the year 2020 [3]. The population-based cancer registries (PBCRs) in India reported that the preventable cancers – breast followed by cervical cancers were the most common sites among females; whereas the mouth was the leading cancer site among males [4]. Furthermore, there was a significant increase in the incidence rate of breast cancer across most of the Indian PBCRs [4]. The Cancer Incidence in Five Continents (CI5) volume XII provided cancer incidence data from India 24 high-quality PBCRs [5]. Based on the CI5 XII database [5], the female breast cancer incidence ranged from 7.2 (Meghalaya) to 45.1 (Bangalore) per 100,000 populations, and for cervix uteri cancer, it ranged from 6.5 (Manipur) to 23.0 (Mizoram) per 100,000 populations. For mouth cancer, among males, it ranged from the lowest 1.5 (Manipur) to the highest 20.7 (Ahmedabad urban); for females, it ranged from 0.7 (Sangrur district) to 9.7 (Meghalaya) per 100,000 populations.

Uttar Pradesh (UP), the most populous state of India, with a total population of 199,812,341 accounts for 16.5% of the total country population. Moreover, UP state is predominantly rural (77.7%), and has 71 districts, more than 310 sub-districts, and over 100,000 villages [6]. With regards to the cancer burden, the state-wise projected cancer cases in India based on the National Cancer Registry Program report for the year 2022 indicated that UP state has the maximum number of cases (14.4%) followed by Maharashtra (8.3%) and West Bengal (7.7%) states [7]. Similarly, compared to other states, UP state had the highest number of deaths due to cancer [7]. Furthermore, Varanasi district PBCR, the first PBCR from UP state, reported that one in 12 men and one in 15 women are at risk of developing cancer in the district. The age-adjusted incidence rate (AAIR) for men and women was 72.1 and 58.4 per 100,000 populations, respectively (year 2018–2019). As per the Varanasi PBCR report, among men, mouth, tongue, gallbladder, lung and liver were the top five leading cancer sites with AAIR of 18.4, 5.6, 4.2, 3.8 and 3.8, respectively. For women, the leading cancer site was the breast, followed by the gall bladder, cervix uteri, ovary and mouth with AAIR of 13.1, 8.2, 7.2, 3.9 and 2.5, respectively [8].

In 2016, the Government of India (GOI) initiated population-based screening programs for common cancers, such as examination of cervix uteri by acetic acid application, breast cancer by clinical examination and oral cancer by visual examination, in an effort to prevent and control cancer. Every 5 years, trained healthcare workers conduct these screening programs for all eligible individuals between the ages of 30 and 65. Under this initiative, screen-positive cases are referred to nearby government health institutions for additional evaluation by a healthcare professional. Managing screen-positive cases further falls under the care of the district and tertiary hospitals [9–11].

The fifth round of the National Family Health Survey (NFHS-5) year 2019–2021, conducted by the GOI, provided data for 724,115 women and 101,839 men [12]. For the first time, state-wise as well as district-wise data on cervical, and breast cancer screening uptake by women and oral cancer screening uptake by both men and women are provided in NFHS-5, and the results are of concern [12, 13]. The spontaneous participation of the target population is one of the critical elements in the success of such nation-wide public health intervention programs [14]. There are several factors that are responsible for the non-compliance to cancer screening examinations, especially for women [15].

As UP state, India has the highest number of cancer cases in the country and NFHS-5 data indicate poor cancer screening uptake, we conducted a secondary data analysis with the aim of providing a district-wise number of women up taking cervical, breast and oral cancer screening residing in UP state. The findings from this research will help in the strategic implementation of public health policies as well as further research on barriers in cancer screening uptake.

## Methods

Under the direction of the Ministry of Health and Family Welfare, GOI, NFHS is executed by coordinating and implementing agencies such as the International Institute for Population Sciences in Mumbai and population survey organisations, which provide valid information on arising health issues of the country. The NFHS-5, the most recent one, was conducted from 17 June 2019 to 30 January 2021. In NFHS-5, first time ever, data on cancer screening participation have been provided. The questions on screening tests/examinations were part of the biomarker questionnaire and were asked to all eligible participating men and women [16]. The following questions were asked in NFHS-5 to obtain information regarding cancer screening uptake among adults [12]:

*To women*

- *Ever undergone a screening test for cervical cancer (%)*
- *Ever undergone a breast examination for breast cancer (%)*
- *Ever undergone an oral cavity examination for oral cancer (%)*

*To men*

- *Ever undergone an oral cavity examination for oral cancer (%)*

**Figure 1.**
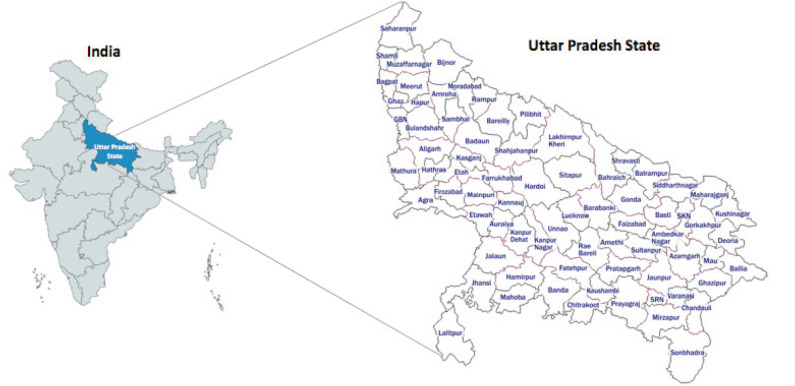
Uttar Pradesh state and its districts.

In NFHS-5, the uptake of cancer screening tests was self-reported by the participants. The uptake was assessed by asking participants if they have ever undergone a screening test. Response to these questions was recorded either in yes or no. For the present paper, we utilised the data from the district-wise NFHS-5 factsheets of UP state, India, and analysed the district-wise NFHS-5 data for cancer screening uptake by adult women (aged 30–49 years). District-level oral cancer screening uptake by men is not on public domain. Based on NFHS-5 data, we estimated number of women who underwent screening in UP state using population projections for the year 2021 provided by the population projections for India and states [17]. The district-wise estimation was done using a projection of the district-level annual population [18]. The number of women from each district of UP state were calculated and are presented here. The UP state along with districts are illustrated in Figure 1.

## Results

The data indicated that in UP state only 1.5% of the total 27,537,000 women population (aged 30–49 years) self-reported having ever had a cervical cancer screening test. Less than one percent of the women self-reported undertaking screening for breast (0.4%) and oral (0.6%) cancer.

The district-wise data for cancer screening in UP state are more worrisome. Some of the districts from UP state did not report a single uptake of screening by women aged 30–49 years. The lowest cervical cancer screening uptake was reported in the Farrukhabad district (0.1%) and the highest in the Deoria district (6.8%). For breast cancer screening, the lowest uptake was reported in Ghazipur, Saharanpur (0.1%) and the highest in Sultanpur (1.8%). For oral cancer screening, the lowest uptake was in Jaunpur (0.1%) to the highest in Unnao (2.4%). The findings are of concern as the population who ever taken cancer screening is very low. The district-wise screening uptake by women in UP state for cervical, breast and oral cancer is presented in Table 1.

## Discussion

The effectiveness of cervical, breast and oral cancer screening methods in cancer prevention and control is well-evident [19], however, the cancer screening uptake in India remains a cause of concern. The present study results indicate that the number of women who attended screening for cervical, breast and oral cancer in different districts of UP state is extremely low. Similar findings were observed in other states of India; the percentage of screening uptake of cervical, breast and oral cancer among women ranged between 0.2%–9.8%, 0.1%–5.6% and 0.2%–7.3%, respectively [13]. The projected cancer incidence cases for UP state indicates the requirement of urgent action especially when the burden of preventable cancers is high.

As per the Indian Census 2011, UP state, India has a predominantly rural population. Women particularly from rural areas face several cultural and social hurdles that are responsible for poor cancer screening compliance. A study conducted to evaluate the rural-urban differences in cancer burden and care in Varanasi district, UP state showed that the incidence and mortality of these common cancers were predominantly higher among rural women [20]. To achieve better adherence to cancer screening in rural areas, it is recommended that communication approaches and delivery strategies that are directed at supporting older, less educated women, who have less interaction with reproductive health services, are needed to further enhance screening uptake [21]. Furthermore, raising cancer awareness among the community along with health-seeking behaviour, and consideration of the sociodemographic specific to the targeted population are crucial in improving cancer screening compliance [22–24]. These factors should be considered in planning of cancer screening program.

The evidence from NFHS-5 regarding poor cancer screening uptake points towards the need to strengthen the ongoing screening and early detection activities, especially at the district level. The GOI is dedicated to delivering services for the prevention of cancer and also, there is an availability of infrastructure and human resources at the district level. For the age group of 30–65, the GOI has started free cervix uteri, breast and oral cancers screening programs. The screening is provided by trained health workers at every 5 years. These population-based screening initiatives started in 2016 and are currently being carried out in many districts [9–11]. Hence, it is recommended that research focusing on exploring reasons for low screening uptake and implementing strategic district-level interventions for improving screening uptake by the population should be prioritised. Factors like inaccessible screening facilities, lack of cancer awareness, prioritised household and work responsibilities as well as day-to-day earnings should be considered when planning for community screening camps for women. Apart from these factors, it should be noted that the Coronavirus disease 2019 pandemic may also have impacted the uptake of screening. Furthermore, the tobacco-attributable cancer burden is high in UP state, India. Policymakers should also focus on the strategic reduction of tobacco use among population as mouth cancer is the leading cancer in both men and women.

The randomised control trials on breast, cervix and oral cancer screening in India have shown better screening compliance [19]; however, it should be noted that these trials are heavily controlled and funded projects and improved compliance is mainly due to provided infrastructure and dedicated human resources. Implementing cancer screening strategies at the community level as a program is still a huge challenge. It is recommended that the policymakers should focus on screening and early detection program for high-risk population at the district level [22]. The steps taken by the GOI are appreciable. Yet, robust efforts are required at the state and district level for improving screening participation as India is witnessing the increasing burden of morbidity and mortality due to cancers. Furthermore, cancer control should be the responsibility of the public health department as well as it is equally the responsibility of the community leaders. Moreover, it is evident that early cancer diagnosis services are better implemented when these programs are integrated with available cancer care facilities, allowing for better monitoring of the programs [22].

There are certain limitations of the study. With regards to the NFHS-5 questionnaire, firstly, the cancer screening-related questions were asked to only 30–49 years of age group women. The age group of 50 and above are not covered under this survey; we recommend adapting the age category based on the current evidence. Secondly, cancer screening-related questions on proforma included medical terms such as ‘cervical’ and ‘oral cavity’ and were used as it is in Hindi language. As there is limited cancer awareness among the rural Indian population [25], the medical terminology used in Hindi language may cause of concern. Furthermore, there is no specific time range mentioned in these questions for cancer screening uptake – for example, the last 5 years. Besides, we could not provide the district-level screening uptake by the men as district-wise data on cancer screening uptake by men was not available in the public domain. However, the overall state data indicated similar results among both populations, with lower oral cancer screening uptake by the men [13]. There are challenges in assessing compliance based on a Demographic Health Survey. The study relies on data from NFHS-5, which is based on surveys. Surveys may have limitations such as recall bias or response bias. Furthermore, the study provided a number of participants based on the population projections, which are based on assumptions and predictions – introducing an element of uncertainty. The accuracy of the projections depends on the reliability of the underlying assumptions.

Besides the survey limitations, the efforts taken by the GOI are appreciable in terms of both providing a framework for population-based common cancer screening as well as cancer screening uptake data through NFHS-5. The questions pertaining to screening uptake might be further refined to enhance comprehension of the obstacles linked to low participation in cancer screening. In future surveys, the following points may be incorporated or added as separate cancer-screening-related questions, including intention to participate, duration of past uptake, any past experience and presentation of symptoms, and any obstacles or reasons that may affect their decision to undergo cervical, breast and oral cancer screenings. Furthermore, the questions should be comprehensible irrespective of participants’ health literacy status.

## Conclusion

The burden of preventable cancer including cervical, breast and oral cancers is a major public health concern in India. The GOI has laid down the guidelines for nationwide screening programs for these common cancers. The study results showed that cancer screening uptake, especially by women, is a cause of concern. Strengthening the existing cancer prevention services should be the foremost priority, especially in resource-limited countries like India. Empowering women by raising cancer awareness in the community, addressing population-specific barriers using innovative and novel approaches as well as implementing comprehensive cancer prevention strategies specific to the local population may improve cancer screening uptake and better cancer control.

## Conflicts of interest

The authors declare that they have no conflicts of interest.

## Funding

This research did not receive any specific grant from funding agencies in the public, commercial, or not-for-profit sectors.

## Ethical approval

None required.

## Author contributions

PC: Writing and reviewing article draft; KP: Conception and design of the study, Statistical analysis; AB: Conception and design of the study, Writing and reviewing manuscript draft, Overall supervision; DS: Reviewing article and providing critical suggestion; PC: Reviewing article and providing critical suggestion; SP: Reviewing article and providing critical suggestion; RD: Reviewing article and providing critical suggestion; RB: Reviewing article and providing critical suggestion, Overall supervision.

## Figures and Tables

**Table 1.** Screening uptake by women aged 30–49 years in UP state and its districts, as per NFHS-5 (2019–2021).

UP/Its districts	Screening uptake by women						Women population (age: 30-49), 2021
	Cervical cancer		Breast cancer		Oral cancer		
	%	*n*	%	*n*	%	*n*	
**UP^#^**	**1.5**	**404,794**	**0.4**	**99,133**	**0.6**	**173,483**	**27,537,000**
Agra (1)	0.3	1,757	0.3	1,757	0.4	2,343	585,732
Aligarh (2)	0.2	955	0.4	1,909	0.0	0	477,303
Allahabad (3) (Currently known as Prayagraj)	1.4	11,075	0.4	3,164	0.7	5,538	791,081
Ambedkar Nagar (4)	4.8	14,918	0.4	1,243	0.4	1,243	310,793
Auraiya (5)	1.4	2,549	0.3	546	0.7	1,275	182,102
Azamgarh (6)	4.6	28,381	1.5	9,255	0.6	3,702	616,983
Baghpat (7)	0.2	310	0.0	0	0.0	0	154,884
Bahraich (8)	0.2	961	0.0	0	1.3	6,247	480,518
Ballia (9)	0.9	3,966	0.4	1,763	0.7	3,085	440,700
Balrampur (10)	3.0	7,722	0.5	1,287	1.6	4,118	257,396
Banda (11)	3.6	8,288	1.4	3,223	1.7	3,914	230,221
Barabanki (12)	1.3	5,198	0.0	0	1.2	4,798	399,842
Bareilly (13)	0.2	1,132	0.0	0	0.0	0	566,025
Basti (14)	3.0	9,110	0.5	1,518	0.0	0	303,669
Bijnor (15)	0.0	0	0.0	0	0.2	934	467,103
Budaun (16) *	0.7	2,935	0.4	1,677	0.2	838	419,244
Bulandshahr (17)	0.0	0	0.2	917	0.0	0	458,515
Chandauli (18)	4.2	10,680	0.8	2,034	0.6	1,526	254,285
Chitrakoot (19)	1.4	1,866	0.4	533	1.3	1,732	133,257
Deoria (20)	6.8	28,428	0.8	3,344	0.5	2,090	418,062
Etah (21)	0.7	1,498	0.3	642	0.0	0	213,961
Etawah (22)	0.2	411	0.0	0	0.2	411	205,532
Faizabad (23)	0.0	0	0.0	0	0.2	632	316,101
Farrukhabad (24)	0.1	228	0.2	457	0.4	914	228,383
Fatehpur (25)	1.9	6,303	1.1	3,649	1.5	4,976	331,713
Firozabad (26)	0.2	652	0.2	652	0.0	0	325,971
Gautam Buddha Nagar (27)	0.2	558	0.0	0	0.2	558	279,010
Ghaziabad (28) *	0.7	5,646	0.0	0	0.9	7,259	806,517
Ghazipur (29)	1.3	6,178	0.1	475	0.2	951	475,260
Gonda (30)	2.1	8,955	0.5	2,132	1.2	5,117	426,430
Gorakhpur (31)	1.8	10,779	1.3	7,785	0.9	5,389	598,819
Hamirpur (32)	2.8	3,650	0.2	261	0.5	652	130,358
Hardoi (33)	0.7	3,526	0.3	1,511	1.5	7,556	503,726
Jalaun (34)	1.4	3,228	0.0	0	0.0	0	230,602
Jaunpur (35)	4.2	25,010	0.0	0	0.1	595	595,484
Jhansi (36)	1.7	4,974	0.5	1463	0.9	2,633	292,561
Jyotiba Phule Nagar (37) Currently known as Amroha	0.0	0	0.2	473	0.2	473	236,551
Kannauj (38)	2.3	4,653	1.0	2,023	1.2	2,428	202,294
Kanpur Dehat (39)	0.6	1,404	0.0	0	0.6	1,404	234,063
Kanpur Nagar (40)	0.8	5,178	0.5	3,236	1.0	6,472	647,226
Kanshiram Nagar (41) Currently known as Kasgani	0.6	999	0.2	333	0.2	333	166,527
Kaushambi (42)	0.5	972	0.2	389	0.6	1,166	194,389
Kheri (43) Currently known as Lakhimpur Kheri	0.6	3,255	0.0	0	0.3	1,627	542,497
Kushinagar (44)	3.0	14,399	0.3	1,440	0.3	1,440	479,963
Lalitpur (45)	0.9	1,433	0.2	318	0.5	796	159,225
Lucknow (46)	1.1	8,083	0.0	0	0.4	2,939	734,855
Mahamaya Nagar (47) Currently known as Hathras	2.4	4,650	1.7	3,294	2.2	4,263	193,766
Mahoba (48)	6.0	7,356	0.7	858	1.8	2,207	122,604
Mahrajganj (49)	0.7	2,549	0.3	1,092	0.3	1,092	364,163
Mainpuri (50)	1.0	2,254	0.4	902	0.0	0	225,422
Mathura (51)	0.7	2,282	0.5	1,630	0.2	652	326,057
Mau (52)	3.7	10,676	1.0	2,885	1.2	3,463	288,549
Meerut (53)	0.2	894	0.0	0	0.0	0	447,058
Mirzapur (54)	3.3	10,503	0.2	637	0.8	2,546	318,277
Moradabad (55) *	0.8	4,883	0.8	4,883	0.4	2,441	610,368
Muzaffarnagar (56) *	0.2	1,062	0.2	1,062	0.2	1,062	531,245
Pilibhit (57)	0.9	2,422	0.2	538	0.4	1,077	269,135
Pratapgarh (58)	0.0	0	0.0	0	0.2	874	437,091
Raebareli (59) *	1.4	6,271	0.2	896	0.7	3,136	447,946
Rampur (60)	0.4	1,159	0.2	579	0.4	1,159	289,741
Saharanpur (61)	0.2	917	0.1	459	0.2	917	458,667
Sant Kabir Nagar (62)	0.8	1,689	0.3	633	0.2	422	211,093
Sant Ravidas Nagar (63)	1.8	3,751	0.0	0	0.6	1,250	208,384
Shahjahanpur (64)	0.2	721	0.2	721	0.2	721	360,638
Shravasti (65)	1.2	1,190	0.4	397	0.5	496	99,184
Siddharth Nagar (66)	2.3	6,714	0.4	1,168	0.2	584	291,901
Sitapur (67)	3.8	21,978	0.2	1,157	0.4	2,313	578,367
Sonbhadra (68)	2.8	7,060	0.0	0	0.2	504	252,130
Sultanpur (69) *	4.3	21,468	1.8	8,987	1.7	8,487	499,253
Unnao (70)	2.0	7,947	0.7	2,781	2.4	9,536	397,351
Varanasi (71)	3.4	17,800	0.9	4,712	1.6	8,377	523,533

# Reference [13]

* Four districts – Sambhal, Hapur Shamli and Amethi were formed after the census year 2011 by dividing Budaun and Moradabad districts; Ghaziabad district; Muzaffarnagar district; and Raebareli and Sultanpur districts respectively
